# Uterine leiomyoma with fumarate hydratase deficiency: A case report

**DOI:** 10.1097/MD.0000000000028142

**Published:** 2021-12-10

**Authors:** Yan Huang, Yan Zhou, Xing Chen, Qin Fang, Huiran Cai, Manxin Xie, Yan Xing

**Affiliations:** a Department of Women Health, Zhangqiu Women and Children Health Hospital, Zhangqiu District, Ji’nan, China; b Department of Obstetrics, The First Affiliated Hospital with Nanjing Medical University, Nanjing, China; c Department of Gynecology, The First Affiliated Hospital with Nanjing Medical University, Nanjing, China.

**Keywords:** cutaneous leiomyomas, fumarate hydratase, leiomyoma, succinate dehydrogenase, type 2 papillary renal cell carcinoma

## Abstract

**Rationale:**

Hereditary leiomyomatosis and renal cell carcinoma is an uncommon autosomal dominant disease caused by mutations in the fumarate hydratase (*FH*) gene. They usually demonstrated multiple uterine myomas and preformed surgical procedures for myomectomy and/or hysterectomy 10 years earlier than sporadic myomas due to early development. This case report describes a woman with multiple uterine leiomyomas diagnosed with *FH* deficiency.

**Patient concerns:**

A 37-year-old woman visited a gynecological clinic for the discovery of uterine leiomyoma for more than 1 year. The size of the largest grew from 42 × 27 × 46 to 98 × 85 × 113 mm in 1 year. She had a history of surgery for breast cancer and thyroid cancer but denied a history of uterine leiomyoma in her family.

**Diagnosis and Interventions:**

The patient underwent successful transabdominal hysterectomy. The pathological results showed multiple uterine leiomyomas (partly cellular leiomyomas) with scattered large bizarre giant cells. Immunohistochemistry results demonstrated *FH* deficiency.

**Outcomes:**

On follow-up, the patient did not have any complications. She was finally referred to the oncologists and urologists for follow-up.

**Lessons:**

Gynecologists should be aware that early onset uterine leiomyoma presenting as large, multiple, and symptomatic lesion, may be associated with *FH* deficiency.

## 1 Introduction

Hereditary leiomyomatosis and renal cell carcinoma (HLRCC), is an uncommon autosomal-dominant disease caused by mutations of fumarate hydratase (*FH*) gene. Patients with HLRCC present with clinical manifestations, including benign uterine leiomyoma, cutaneous leiomyomas, and type 2 papillary renal cell carcinoma.^[1]^ For female patients with HLRCC, it is estimated that up to 80% to 100% of individuals are diagnosed with benign uterine leiomyoma. They usually demonstrated multiple uterine myomas and require surgical procedures like myomectomy and/or hysterectomy 10 years earlier than sporadic myomas due to early development.^[2]^ We reported a 37-year-old woman suffering from uterine leiomyoma and the pathology demonstrated FH negative uterine leiomyoma.

## 2 Case presentation

**Figure 1:**
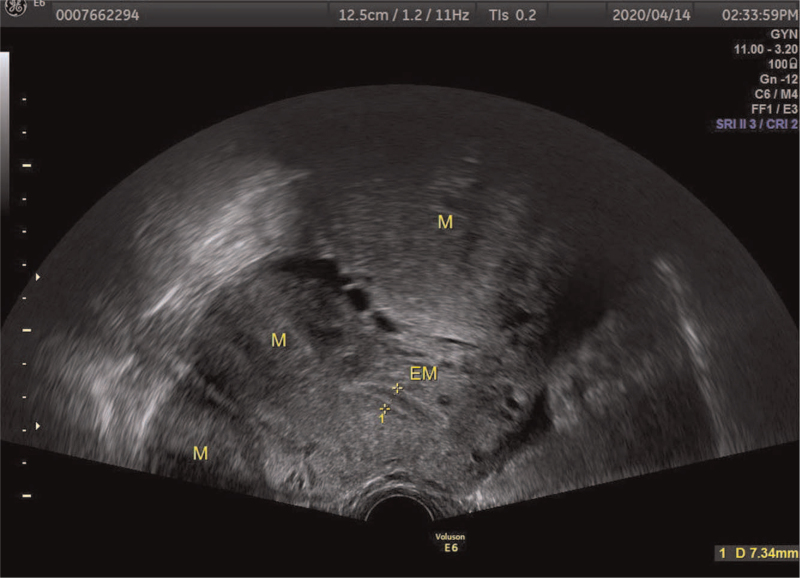
Multiple uterine leiomyomas by pelvic ultrasound. The size of the largest one was approximately 98 × 85 × 113 mm.

**Figure 2:**
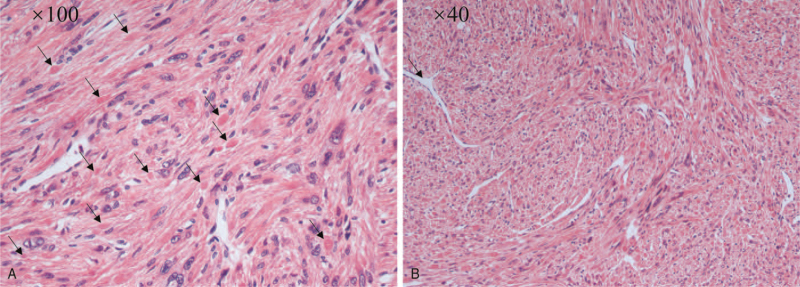
Morphological characteristics of uterine leiomyoma with fumarate hydratase deficiency. (A) Cytoplasmic eosinophilic globules (arrow) (×100). (B) Staghorn vessels (arrow) (×40).

**Figure 3:**
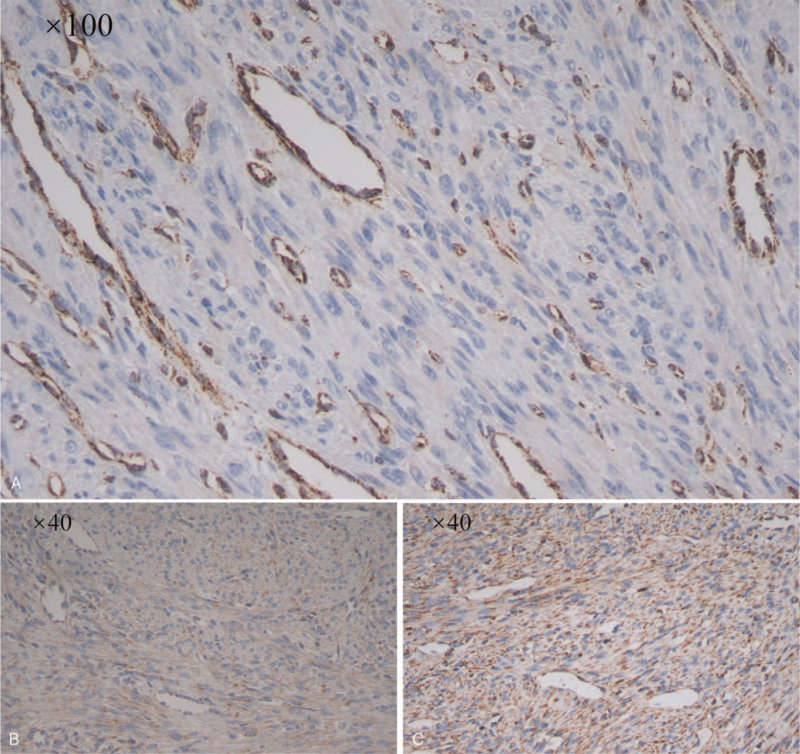
Results of immunohistochemistry of uterine leiomyoma with fumarate hydratase (FH) deficiency. (A) Cells of uterine leiomyoma were negative to FH, while adjacent vascular endothelial cells and vascular smooth muscle cells were positive to FH (showed brown). Further positive results of immunohistochemistry of succinate dehydrogenase (SDH) subunit A (SDHA) (B) and subunit B (SDHB) (C) confirmed the deficiency of FH.

A 37-year-old woman visited the gynecological clinic for an incidental finding of uterine leiomyoma for more than 1-year duration. In November 2018, multiple uterine leiomyomas were observed on ultrasound, and the largest leiomyoma was approximately 42 × 27 × 46 mm. She underwent regular follow-up for uterine leiomyomas. She underwent pelvic ultrasound again in April 2020 and found a significant increase in the diameter of the myoma, with the largest diameter being approximately 98 × 85 × 113 mm (Fig. 1). Physical examination revealed a huge mass palpable to the level of the umbilicus. No obvious abnormality was found on ultrasonography of the urinary system. She had a history of breast cancer and thyroid cancer surgery, as well as chemotherapy for breast cancer. She denied a history of uterine leiomyoma in her family. On suspicion of uterine leiomyosarcoma for rapid increase of the tumor, she underwent transabdominal hysterectomy successfully. Results of hematoxylin and eosin (H&E) staining showed multiple uterine leiomyomas (partly cellular leiomyomas) with scattered large bizarre giant cells. Cytoplasmic eosinophilic globules and staghorn vessels were also observed (Fig. 2). Immunohistochemistry results demonstrated that cells of uterine leiomyoma were negative for FH, while adjacent vascular endothelial cells and vascular smooth muscle cells were positive for FH. Further positive immunohistochemistry results for succinate dehydrogenase (SDH) subunit A (SDHA) and subunit B (SDHB) confirmed FH deficiency (Fig. 3). Other characteristics of uterine leiomyoma cells, including estrogen receptor (+), progesterone receptor (+), p53(+), H-caldesmon (+), smooth muscle actin (SMA) (+), CD31(+), CD10(−), and Ki-67(5%+) were also verified. Based on the combined results of immunohistochemistry and H&E staining, the woman was finally diagnosed with uterine leiomyoma with *FH* deficiency. On a follow-up visit, the patient was well without any discomfort. She was finally referred to the oncologists and urologists for follow-up.

## 3 Discussion

The protein encoded by *FH*, which catalyzes fumarate to malate, is one of the most important elements in the tricarboxylic acid cycle (Krebs cycle) enzyme. Mutations in *FH* lead to reduced enzyme activity and accumulation of fumarate, further causing dysfunction of mitochondrial respiratory chain activity.^[3]^ Furthermore, accumulated fumarate could improve the expression of hypoxia-inducible factor 1α and nuclear factor (erythroid-2) like-2 transcription factor, which participate in various pathophysiological processes such as angiogenesis, epithelial-to-mesenchymal transition, and cancer progression.^[4]^ Furthermore, FH has also been recognized as a suppressor of homologous recombination DNA repair, thus compromising genome integrity.^[5]^

Nearly all women with HLRCC have uterine leiomyoma, which is early onset, multiple, large, and symptomatic, ultimately resulting in early myomectomy or hysterectomy. However, the pathological examination results are typically benign.^[6]^ Although uterine leiomyoma could be derived from mutations in *the FH* gene, a sporadic somatic mutation of the *FH* gene, which is not correlated with HLRCC, was more frequent in the clinic.^[7]^*FH* deficiency can be observed in various types of uterine leiomyomas, especially in myomas with bizarre nuclei, but is also distributed in cellular and conventional leiomyomas.^[8]^ A study of 108 cases of leiomyoma with bizarre nuclei demonstrated that 62% (67/108) of cases had loss of *FH* expression, while only 0.2% (1/50) in usual uterine leiomyoma and none (0/42) in leiomyosarcomas,^[9]^ suggesting that leiomyoma with bizarre nuclei was an important pathological characteristic of FH-deficient uterine leiomyoma. Bennett et al^[10]^ summarized the morphologic features of 31 cases of *FH (−)* uterine leiomyoma and found that they often had staghorn vessels, scattered bizarre nuclei, eosinophilic cytoplasmic inclusions, prominent eosinophilic nucleoli, and perinucleolar halos. Siegler et al^[11]^ summarized 22 *FH* (− uterine leiomyomas that were diagnosed by immunohistochemistry of FH and found that the median age was 36. At least 18 (81.8%) patients had multiple leiomyomas (ranging from 2 to 14 cm), with a maximum diameter 11.8 cm. It was estimated that approximately 0.4% of uterine leiomyomas were FH-deficient.^[11]^ However, none of them had a family history of HLRCC. Among these patients, 1 had adenoma of the thyroid, 1 was diagnosed with colorectal adenocarcinoma and endometrioid carcinoma, and 1 had a history of breast cancer. In our case, the patient had uterine leiomyoma at the age of 35 years and underwent hysterectomy at 37. Pathological examination showed multiple, cellular, and bizarre nucleus leiomyomas, as well as cytoplasmic eosinophilic globules and staghorn vessels, which were consistent with previously reported morphological characteristics of *FH-*deficient uterine leiomyomas. Although there were no clinical findings of HLRCC, the patient had previously suffered from thyroid and breast cancer. However, the relationship between these 2 cancers and HLRCC remains unknown.

Cutaneous leiomyoma manifested as tan to reddish papules or nodules of different sizes (2–40 mm), which mainly occurred on the limbs and trunk or back.^[11]^ Histopathological examination demonstrated the classic characteristics of well-differentiated smooth muscle cells. It occurs in 76% to 100% of patients with HLRCC, with an average morbidity age of 25. Furthermore, 60% to 100% carriers of FH mutation older than 40 years suffer from cutaneous leiomyomas.^[12]^ The onset of cutaneous leiomyoma was earlier 6 years (25 vs 31) than uterine leiomyoma at an average. However, approximately 40% of cutaneous leiomyomas have no obvious clinical manifestations, leading to the late detection of genetic abnormalities.^[13]^

Type 2 papillary renal cell carcinoma, which is an aggressive and refractory tumor, is the most frequent renal neoplasm in patients with HLRCC. For HLRCC patients, the prevalence of this carcinoma is reported to be 6.5-fold higher than that in the general population. It was recognized as a distinct renal tumor subtype called HLRCC-related renal cell carcinoma according to the World Health Organization genitourinary cancer classification in 2016. The incidence of type 2 papillary renal cell carcinoma in patients with HLRCC ranges from 6% to 62%. Other types of renal cell carcinoma, such as collecting ducts and clear cell cancers, were also found in HLRCC patients. Unlike uterine leiomyomas, renal tumors are often solitary and unilateral, with a mean onset age of 40 years. It is estimated that the lifelong risk of renal cancer in patients with HLRCC is approximately 15%.^[1,2,11,12]^

In addition to the above clinical manifestations, mutations in *FH* genes are predisposed to malignant pheochromocytomas and paragangliomas.^[12]^ Furthermore, FH variants also account for fumaric aciduria, which is an autosomal recessive disorder characterized by brain malformations, developmental delay, and seizures, leading to early neurological impairment.

Uterine leiomyomas in patients with FH deficiency can be initially presumed by routine H&E staining due to its distinctive histological characteristics. Immunohistochemistry of FH has been widely applied for the identification of FH deficiency. Fumarate also succinates cysteine to S-(2-succinyl) cysteine, thus making it a potential biomarker of FH pathogenic variants. However, the antibody of S-(2-succinyl) cysteine is currently commercially unavailable and cannot be used for clinical diagnosis.^[14]^ SDH, especially SDHB, was applied as a control to verify the results of FH loss.^[11]^ In our case, immunohistochemistry of SDHA and SDHB was performed and was positive, further confirming the characteristics of FH deficiency in cells of uterine leiomyoma. The absence of FH immunohistochemical staining in uterine leiomyoma cells suggests *FH* mutation. However, immunohistochemistry is not absolutely sensitive and specializes in *FH* deficiency. It has been reported that more than 130 different germline mutations, including missense, frameshift, indel, splice site, and nonsense mutations, are involved in HLRCC patients.^[6]^ For example, missense mutations in the *FH* could lead to the expression of nonfunctional proteins that are findable by immunohistochemical staining, leading to false positive results in FH.^[15]^ As a result, molecular genetic testing is still the gold standard to confirm HLRCC.

## 4 Conclusion

Uterine leiomyomas with *FH* deficiency are rare. Although cutaneous and uterine leiomyomas in HLRCC patients are mostly benign, renal cell carcinoma is always aggressive, and the prognosis is poor. For individuals with suspected clinical manifestations and positive genetic testing, it is highly recommended to refer to oncologists and urologists for follow-up.

## Acknowledgments

The authors thank the pathologists of the First Affiliated Hospital with Nanjing Medical University for offering images.

## Author contributions

**Conceptualization:** Xing Chen, Yan Xing.

**Data curation:** Qin Fang, Huiran Cai, Manxin Xie.

**Formal analysis:** Qin Fang, Huiran Cai, Manxin Xie.

**Supervision:** Xing Chen, Yan Xing.

**Writing – original draft:** Yan Huang, Yan Zhou.

**Writing – review & editing:** Xing Chen, Yan Xing.
